# Synthesis and crystal structure of bis­[μ-*N*,*N*-bis­(2-amino­eth­yl)ethane-1,2-di­amine]­bis­[*N*,*N*-bis­(2-amino­eth­yl)ethane-1,2-di­amine]-μ_4_-oxido-hexa-μ_3_-oxido-octa-μ_2_-oxido-tetra­oxido­tetra­nickel(II)hexa­tantalum(V) nona­deca­hydrate

**DOI:** 10.1107/S2056989021011531

**Published:** 2021-11-09

**Authors:** Dana-Céline Krause, Christian Näther, Wolfgang Bensch

**Affiliations:** a Institut für Anorganische Chemie, Christian-Albrechts-Universität zu Kiel, Max-Eyth-Str. 2, D-24118 Kiel, Germany

**Keywords:** crystal structure, layered structure, polyoxidotantalate, Lindqvist-type anion

## Abstract

Reaction of K_8_{Ta_6_O_19_}·16H_2_O with [Ni(tren)(H_2_O)Cl]Cl·H_2_O in different solvents led to the formation of single crystals of the title compound, [Ni_4_Ta_6_O_19_(C_6_H_18_N_4_)_4_]·19H_2_O or {[Ni_2_(κ^4^-tren)(μ-κ^3^-tren)]_2_Ta_6_O_19_}·19H_2_O (tren is *N*,*N*-bis­(2-amino­eth­yl)-1,2-ethanediamine, C_6_H_18_N_4_). In its crystal structure, one Lindqvist-type anion {Ta_6_O_19_}^8–^ (point group symmetry  ) is connected to two Ni^II^ cations, with both of them coordinated by one tren ligand into discrete units. Both Ni^II^ cations are sixfold coordinated by O atoms of the anion and N atoms of the organic ligand, resulting in slightly distorted [NiON_5_] octa­hedra for one and [NiO_3_N_3_] octa­hedra for the other cation. These clusters are linked by inter­molecular O—H⋯O and N—H⋯O hydrogen bonding involving water mol­ecules into layers parallel to the *bc* plane. Some of these water mol­ecules are positionally disordered and were refined using a split model. Powder X-ray diffraction revealed that a pure crystalline phase was obtained but that on storage at room-temperature this compound decomposed because of the loss of crystal water mol­ecules.

## Abstract 2

The crystal structure of the title compound consists of discrete {[Ni_2_(κ^4^-tren)(μ- κ^3^-tren)]_2_Ta_6_O_19_} cluster mol­ecules that are linked by inter­molecular O—H⋯O and N—H⋯O hydrogen bonding into layers extending parallel to the *bc* plane.

## Chemical context

The investigation of synthesis conditions and crystal structures of new inorganic–organic hybrid polyoxidometalates (POMs) of V, Nb, Ta, Mo or W is still an emerging research field in inorganic chemistry. The enormous variety of their structural, physical and chemical properties and the resulting potential applications are reflected in the large number of reported compounds (Tagliavini *et al.*, 2021 ▸; Streb, 2012 ▸; Bijelic *et al.*, 2019 ▸; Yamase, 2013 ▸; König, 2020 ▸; Čolović *et al.*, 2020 ▸; Monakhov *et al.*, 2015 ▸). Within the POM family, polyoxidoniobates and -tantalates have a special position because of their challenging synthesis conditions, *i.e*. high pH values are required as a result of the high stability of their respective oxides. This is the reason why we have been engaged in the research field of POM chemistry for several years, with the aim in developing new synthesis routes, also with an increasing focus on the PONb and POTa chemistry (Müscher-Polzin *et al.*, 2020*a* ▸,*b* ▸; Dopta *et al.*, 2018*a* ▸,*b* ▸, 2020 ▸). Most of the POMs are usually synthesized by solvothermal reactions using slightly soluble metal oxides. It turned out that the use of water-soluble compounds as precursor materials is more effective for generating new compounds, which opens the possibility of developing more efficient syntheses at room temperature (Dopta *et al.*, 2020 ▸; Mahnke *et al.*, 2018*a* ▸,*b* ▸). Some transition metal (TM) decorated POTas have also been synthesized by slow crystallization at room temperature (Guo *et al.*, 2011 ▸; Li *et al.*, 2019 ▸), which is characterized by long reaction times and high sensibility for parameter changes during reaction. To overcome these drawbacks, we were inter­ested in the possibility of faster crystallization times. To achieve this goal, we used preformed TM complexes and a special combination of different solvent gradients in the reaction vessel. Appropriate TM complexes are based on the tetra­dentate ligand *N*,*N*-bis­(2-amino­eth­yl)-1,2-ethanedi­amine (tren), which offers coordination flexibility, providing two free coordination sites in an octa­hedral environment, with the possibilities for further ligation to O atoms of POMs or acting as charge-balancing cations. Based on that reasoning, an aqueous solution of K_8_[Ta_6_O_19_]·16H_2_O was reacted with the preformed complex [Ni(tren)(H_2_O)Cl]Cl·H_2_O at room temperature, leading to crystallization of violet needle-like crystals of the title compound, which was characterized by single-crystal X-ray diffraction. Comparison of the experimental powder X-ray diffraction pattern with that calculated from single crystal data revealed that a pure crystalline phase had formed. However, the relatively high background indicated the presence of some amount of an amorphous phase (see Fig. S1 in the supporting information). This is in line with the observation that the title compound is very unstable in air, which might be traced back to the loss of crystal water mol­ecules, and was the reason why further investigations were not performed. 
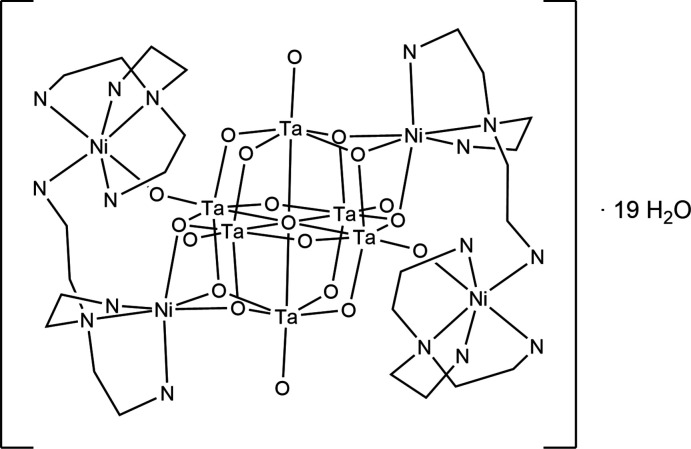


## Structural commentary

**Figure 1:**
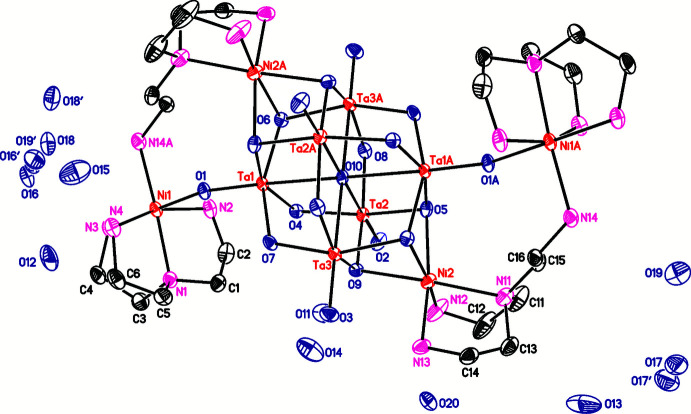
The mol­ecular entities in the crystal structure of the title compound with displacement ellipsoids drawn at the 50% probability level. Hydrogen atoms were omitted for clarity. [Symmetry code: (A) −*x* + 1, −*y*, −z + 2].

The crystal structure of {[Ni_2_(κ^4^-tren)(μ-κ^3^-tren)]_2_Ta_6_O_19_}·19H_2_O consists of one Lindqvist-type anion {Ta_6_O_19_}^8–^, located on a center of inversion, as well as two Ni^II^ cations, two *N*,*N*-bis­(2-amino­eth­yl)-1,2-ethanedi­amino ligands and nineteen water mol­ecules that are located in general positions (Figs. 1 ▸ and 2 ▸). Some of the water O atoms are positionally disordered and were refined using a split model without locating their attached hydrogen atoms.

**Figure 3:**
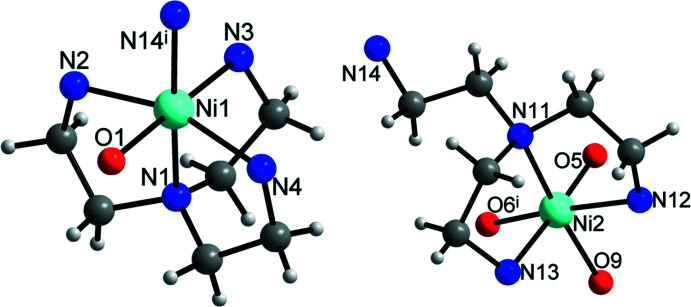
View of the coordination environments of the two Ni^II^ cations with labeling of selected atoms. H atoms bonded to N atoms were omitted for clarity. [Symmetry code: (i) −*x* + 1, −*y*, −*z* + 2.]

The {Ta_6_O_19_}^8–^ anion is composed of six TaO_6_ octa­hedra sharing common edges. The Ta—O bond lengths range from 1.786 (2) to 2.057 (2) Å, which is consistent with common values. Bond-valence-sum calculations (Brown & Altermatt, 1985 ▸; Liu & Thorp, 1993 ▸; O’Keefe & Brese, 1991 ▸) led to values of 4.98 valence units (v.u.) for Ta1, of 1.78 v.u. for Ni1 and of 1.69 v.u. for Ni2, which is in reasonable agreement with the oxidation states of +5 and +2 for Ta and Ni, respectively. Two symmetry-related pairs of Ni^II^ cations are covalently attached to the {Ta_6_O_19_}^8–^ core: Ni2 forms bonds to three μ_2_-bridging O atoms with Ni—O bond lengths between 2.103 (2) and 2.170 (2) Å, while Ni1 is attached to a terminal O atom with a Ni—O bond length of 2.072 (2) Å (Fig. 3 ▸, Table 1 ▸), which is slightly larger than the sum of their ionic radii (Ni^II^ with CN6 = 0.69 Å, O^2−^ = 1.35 Å; Shannon, 1976 ▸). The Ni1 cation is further coordinated by four N donor atoms (N1–N4) of one tren ligand and an additional N atom (N14) of another tren ligand, with Ni—N bonds ranging from 2.076 (3) to 2.172 (3) Å (Table 1 ▸), which is in agreement with reported values of similar structures (Dopta *et al.* 2018*a* ▸; Hegetschweiler *et al.*, 2002 ▸; Niu *et al.*, 2011 ▸; Kim *et al.*, 2004 ▸; Mash *et al.*, 2019 ▸; Junk & Steed, 2007 ▸). One tren ligand connects both Ni^II^ cations *via* an Ni—μ-N—Ni bond of 2.082 (3) Å. Both Ni^II^ cations are in an octa­hedral environment, resulting in [Ni2O_3_N_3_] and [Ni1ON_5_] units (Fig. 3 ▸). The bond angles within the complexes cover a wide range between 82.40 (13) and 178.92 (11)° for [Ni2O_3_N_3_] and between 74.98 (9) and 174.07 (11)° for [Ni1ON_5_], which shows that both Ni^II^ cations have a distorted octa­hedral environment. The distortion is caused by steric demands, because both Ni^II^ cations are coordinated by the anionic cluster as well as by tren ligands.

## Supra­molecular features

**Figure 4:**
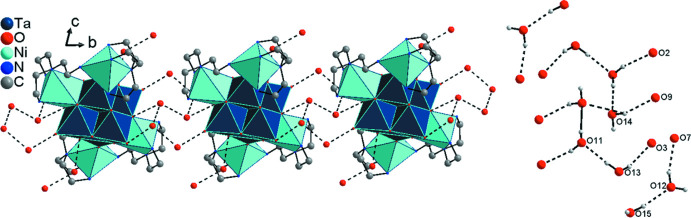
View of the hydrogen-bonded chains running parallel to [010]. Inter­molecular hydrogen bonding is indicated by dashed lines. In the left part, hydrogen atoms were omitted for clarity.

**Figure 5:**
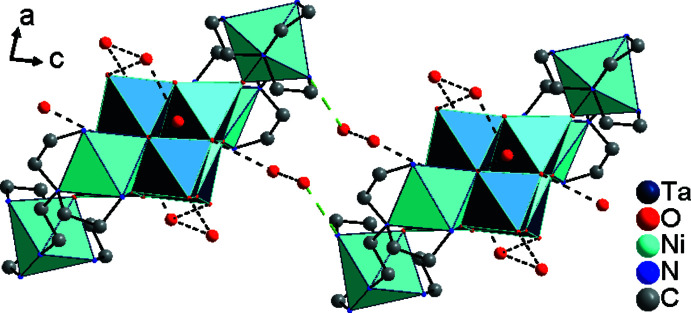
View of the hydrogen-bonded layer extending parallel to the *bc* plane by linking the [010] chains *via* O_water_⋯N bonds (black dashed lines: O_water_⋯O_water_; green dashed lines: O_water_⋯N). Hydrogen atoms were omitted for clarity.

In the crystal, the discrete mol­ecular moieties are linked by O—H⋯O and O—H⋯N hydrogen bonds between the crystal water mol­ecules and the O atoms of the {Ta_6_O_19_}^8–^ core (Table 2 ▸). The water mol­ecules form discrete units categorized as *D*6 (Infantes *et al.*, 2003 ▸; Infantes & Motherwell, 2002 ▸), of which each water mol­ecule is attached to an O_cluster_ atom with O_cluster_⋯O distances between 1.88 and 1.99 Å and condensed into chains extending parallel to [010] (Fig. 4 ▸). The [010] chains are further linked by O_water_—H⋯N bonds with O⋯N separations between 2.232 and 2.537 Å, yielding another chain that propagates parallel to [001] (Table 2 ▸), finally forming a layered structure parallel to the *bc* plane (Fig. 5 ▸). There are additional C—H⋯N inter­actions (Table 2 ▸). From both the C⋯N distances and the angles, it is obvious that these represent only weak inter­actions.

## Database survey

There are only a few crystal structures of POMs reported in the literature with [Ni^II^(tren)_*x*_] complexes covalently attached to the anionic core. Our group has already reported the rare [Ni_2_(tren)_3_]^4+^ and [{Ni(tren)}(trenH_2_){Ni(tren)}]^6+^ complexes that act as linking units between several anionic moieties (Lühmann *et al.*, 2014 ▸; Wang *et al.*, 2013 ▸). In these structures, the Ni^II^ cation is coordinated by one tetra­dentate ligand and one additional tren mol­ecule connecting two Ni^II^ cations of neighboring POV ({V_15_Ge_6_}) clusters. A connection of two Ni^II^ cations bonded to separated clusters *via* two tren mol­ecules (with κ^3^ and κ^4^ modes) has not been reported until now. However, the crystal structure of a similar complex, *viz*. [Ni_3_((μ-*tren*)_2_(*tren)*_2_(H_2_O)_2_]^6+^ was reported previously (Matelková *et al.*, 2013 ▸).

## Synthesis and crystallization

**Synthesis**

All chemicals except K_8_{Ta_6_O_19_}·16H_2_O were purchased from commercial sources and were used without further purification [*N*,*N*-bis­(2-amino­eth­yl)-1,2-ethanedi­amine (tren) >96%, Aldrich; Ta_2_O_5_ 99% Ta, Alfa Aesar; NiCl_2_·6H_2_O > 97%, Merck; KOH 85%, abcr; di­methyl­sulfoxide (DMSO) 99%, Grüssing]. The water-soluble precursor K_8_{Ta_6_O_19_}·16H_2_O was prepared according to Filowitz *et al.* (1969 ▸), and the prefabricated complex [Ni(tren)(H_2_O)Cl]Cl·H_2_O using the protocol of Marzotto *et al.* (1993 ▸).

0.03 mmol of Ni[(tren)(H_2_O)Cl]Cl·H_2_O were dissolved in 1 ml of a 4:1 DMSO:water solution (*v*/*v*) and subsequently transferred into a 5 ml snap-cap glass tube. Then 1 ml of a 3:1 mixture (*v*/*v*) of DMSO and water and a solution of 0.0125 mmol of K_8_{Ta_6_O_19_}·16H_2_O in 1 ml of water (pH = 12.3) were added slowly, one after the other, into the tube, which then was closed and left at room temperature. After a few days, pink–violet needle-shaped crystals were filtered off and washed with mother liquor.

**Experimental details**

The PXRD measurement was performed with Cu *K*α_1_ radiation (λ = 1.540598 Å) using a Stoe Transmission Powder Diffraction System (STADI P) equipped with a MYTHEN 1K detector and a Johansson-type Ge(111) monochromator.

## Refinement

Crystal data, data collection and structure refinement details are summarized in Table 3 ▸. The C- and N-bound hydrogen atoms were refined with idealized positions with *U*_iso_(H) = 1.2*U*_eq_(C,N) using a riding model. Some of the hydrogen atoms belonging to water mol­ecules were located in a difference-Fourier map. Their bond lengths were set to ideal values and they were refined with *U*_iso_(H) = 1.5*U*_eq_(O). Some of the water atoms (O16–O19) are positionally disordered and were refined using a split model with 50% occupation for each of the corresponding sites; O20 was refined with one position and an occupation of 50%. The hydrogen atoms of water mol­ecules that could not be located were considered in the calculation of the mol­ecular formula.

## Supplementary Material

Crystal structure: contains datablock(s) I. DOI: 10.1107/S2056989021011531/wm5616sup1.cif

Structure factors: contains datablock(s) I. DOI: 10.1107/S2056989021011531/wm5616Isup2.hkl

Figure S1: Experimental and calculated XRPD powder pattern of the title compound. DOI: 10.1107/S2056989021011531/wm5616sup3.tif

CCDC reference: 2119604

Additional supporting information:  crystallographic information; 3D view; checkCIF report

## Figures and Tables

**Figure 2:**
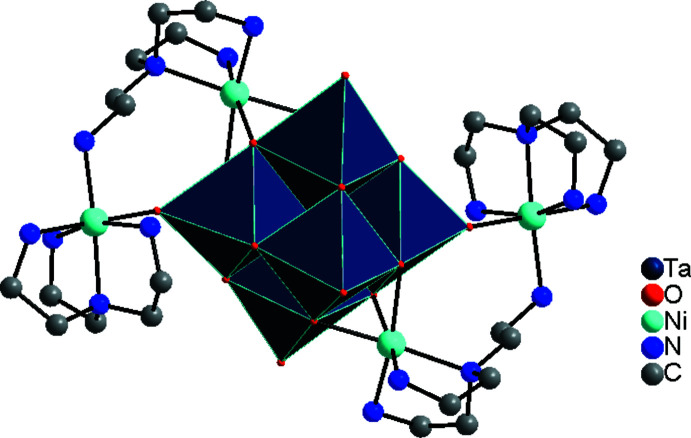
View of the cluster motif of the title compound. Hydrogen atoms were omitted for clarity.

**Table 1:** Selected bond lengths (Å)

O1—Ni1	2.072 (2)	Ni1—N3	2.105 (3)
O5—Ni2	2.170 (3)	Ni1—N4	2.139 (3)
O6—Ni2^i^	2.149 (2)	Ni1—N14^i^	2.082 (3)
O9—Ni2	2.103 (2)	Ni2—N11	2.172 (3)
Ni1—N1	2.111 (3)	Ni2—N12	2.094 (4)
Ni1—N2	2.120 (3)	Ni2—N13	2.076 (3)

Symmetry code: (i) -x+1, -y, -z+2.

**Table 2:** Hydrogen-bond geometry (Å, °)

*D*—H⋯*A*	*D*—H	H⋯*A*	*D*⋯*A*	*D*—H⋯*A*
C1—H1*B*⋯O4	0.99	2.52	3.293 (5)	135
N2—H2*C*⋯O4^ii^	0.91	2.59	3.394 (4)	148
N2—H2*C*⋯O8^ii^	0.91	2.42	3.208 (4)	146
N2—H2*D*⋯O4	0.91	2.59	3.236 (4)	129
N2—H2*D*⋯N2^ii^	0.91	2.62	3.338 (6)	136
C3—H3*A*⋯O19′^iii^	0.99	2.53	3.253 (8)	129
C3—H3*B*⋯O13^iv^	0.99	2.64	3.301 (6)	124
C4—H4*B*⋯O19′^iii^	0.99	2.60	3.135 (7)	114
N3—H3*C*⋯O18	0.91	2.40	3.197 (7)	146
N3—H3*C*⋯O19′	0.91	2.45	3.266 (8)	149
N3—H3*D*⋯O8^ii^	0.91	2.02	2.915 (4)	169
N4—H4*C*⋯O15	0.91	2.54	3.244 (5)	135
C11—H11*B*⋯O16′^ii^	0.99	2.57	3.497 (12)	156
C12—H12*B*⋯O20	0.99	2.47	2.977 (8)	111
N12—H12*C*⋯O20	0.91	2.33	2.995 (7)	130
N12—H12*D*⋯O2	0.91	2.16	2.951 (4)	145
N12—H12*D*⋯O18′^ii^	0.91	2.39	3.168 (8)	144
C13—H13*A*⋯O13	0.99	2.59	3.380 (6)	137
N13—H13*C*⋯O3	0.91	2.10	2.937 (5)	153
N13—H13*D*⋯O14^iv^	0.91	2.23	3.103 (5)	160
C15—H15*A*⋯O1^i^	0.99	2.45	3.105 (5)	124
C16—H16*A*⋯O6^i^	0.99	2.63	3.514 (4)	149
C16—H16*B*⋯O11^v^	0.99	2.58	3.400 (5)	140
N14—H14*C*⋯O19	0.91	2.62	3.447 (7)	151
N14—H14*C*⋯O19′^i^	0.91	2.27	3.082 (7)	148
N14—H14*D*⋯O2^v^	0.91	2.04	2.941 (4)	169
O11—H11*C*⋯O2	0.84	1.97	2.794 (4)	165
O11—H11*D*⋯O14	0.84	2.00	2.826 (5)	170
O12—H12*E*⋯O7^vi^	0.84	1.97	2.784 (4)	163
O12—H12*F*⋯O16	0.84	1.86	2.686 (11)	170
O12—H12*F*⋯O16′	0.84	2.25	3.079 (11)	169
O13—H13*E*⋯O11^v^	0.84	1.89	2.696 (5)	161
O13—H13*F*⋯O3^vii^	0.84	1.93	2.698 (4)	152
O13—H13*F*⋯O20^v^	0.84	2.59	3.101 (7)	120
O14—H14*E*⋯O20^iv^	0.84	1.94	2.757 (7)	164
O14—H14*F*⋯O9	0.84	2.01	2.762 (4)	149
O15—H15*C*⋯O17^viii^	0.84	2.01	2.787 (9)	153
O15—H15*C*⋯O17′^viii^	0.84	2.02	2.850 (10)	170
O15—H15*D*⋯O12^iii^	0.84	1.90	2.723 (6)	168

Symmetry codes: (i) -x+1, -y, -z+2; (ii) -x+2, -y, -z+2; (iii) -x+2, -y, -z+3; (iv) -x+1, -y+1, -z+2; (v) x-1, y, z; (vi) x+1, y, z; (vii) -x, -y+1, -z+2; (viii) x+1, y-1, z+1.

**Table 3:** Experimental details

Crystal data	
Chemical formula	[Ni_4_Ta_6_O_19_(C_6_H_18_N_4_)_4_]·19H_2_O
*M*_r_	2551.81
Crystal system, space group	Triclinic, *P*\overline{1}
Temperature (K)	100
*a*, *b*, *c* (Å)	10.5033 (1), 12.0980 (2), 13.8640 (2)
α, β, γ (°)	73.748 (1), 80.918 (1), 80.842 (1)
*V* (Å^3^)	1657.76 (4)
*Z*	1
Radiation type	Mo *K*α
μ (mm^−1^)	11.06
Crystal size (mm)	0.11 × 0.06 × 0.01 × 0.02 (radius)

Data collection	
Diffractometer	XtaLAB Synergy, Dualflex, HyPix
Absorption correction	Multi-scan (*CrysAlis PRO*; Rigaku OD, 2021 ▸)
*T*_min_, *T*_max_	0.686, 0.694
No. of measured, independent and observed [*I* > 2σ(*I*)] reflections	44996, 7897, 7404
*R*_int_	0.030
(sin θ/λ)_max_ (Å^−1^)	0.658

Refinement	
*R*[*F* ^2^ > 2σ(*F* ^2^)], *wR*(*F* ^2^), *S*	0.021, 0.059, 1.05
No. of reflections	7897
No. of parameters	439
H-atom treatment	H-atom parameters constrained
Δρ_max_, Δρ_min_ (e Å^−3^)	2.83, −1.13

Computer programs: *CrysAlis PRO* (Rigaku OD, 2021 ▸), *SHELXT* (Sheldrick, 2015*a* ▸), *SHELXL* (Sheldrick, 2015*b* ▸), *DIAMOND* (Brandenburg & Putz, 1999 ▸) and *publCIF* (Westrip, 2010 ▸).

## supplementary crystallographic information

### Crystal data

**Table:**

[Ni_4_Ta_6_O_19_(C_6_H_18_N_4_)_4_]·19H_2_O	*Z* = 1
*M**_r_* = 2551.81	*F*(000) = 1210
Triclinic, *P*1	*D*_x_ = 2.556 Mg m^−^^3^
*a* = 10.5033 (1) Å	Mo *K*α radiation, λ = 0.71073 Å
*b* = 12.0980 (2) Å	Cell parameters from 37095 reflections
*c* = 13.8640 (2) Å	θ = 2.4–34.0°
α = 73.748 (1)°	µ = 11.06 mm^−^^1^
β = 80.918 (1)°	*T* = 100 K
γ = 80.842 (1)°	Needle, light violet
*V* = 1657.76 (4) Å^3^	0.11 × 0.06 × 0.01 × 0.02 (radius) mm

### Data collection

**Table:**

XtaLAB Synergy, Dualflex, HyPix diffractometer	7897 independent reflections
Radiation source: micro-focus sealed X-ray tube, PhotonJet (Mo) X-ray Source	7404 reflections with *I* > 2σ(*I*)
Mirror monochromator	*R*_int_ = 0.030
Detector resolution: 10.0000 pixels mm^-1^	θ_max_ = 27.9°, θ_min_ = 2.4°
ω scans	*h* = −13→13
Absorption correction: multi-scan (CrysAlisPro; Rigaku OD, 2021)	*k* = −15→15
*T*_min_ = 0.686, *T*_max_ = 0.694	*l* = −18→18
44996 measured reflections	

### Refinement

**Table:**

Refinement on *F*^2^	Primary atom site location: dual
Least-squares matrix: full	Hydrogen site location: mixed
*R*[*F*^2^ > 2σ(*F*^2^)] = 0.021	H-atom parameters constrained
*wR*(*F*^2^) = 0.059	*w* = 1/[σ^2^(*F*_o_^2^) + (0.0386*P*)^2^ + 3.1986*P*] where *P* = (*F*_o_^2^ + 2*F*_c_^2^)/3
*S* = 1.05	(Δ/σ)_max_ = 0.003
7897 reflections	Δρ_max_ = 2.83 e Å^−^^3^
439 parameters	Δρ_min_ = −1.13 e Å^−^^3^
0 restraints	

### Special details

**Table:**

Geometry. All esds (except the esd in the dihedral angle between two l.s. planes) are estimated using the full covariance matrix. The cell esds are taken into account individually in the estimation of esds in distances, angles and torsion angles; correlations between esds in cell parameters are only used when they are defined by crystal symmetry. An approximate (isotropic) treatment of cell esds is used for estimating esds involving l.s. planes.

### Fractional atomic coordinates and isotropic or equivalent isotropic displacement parameters (Å^2^)

**Table:**

	*x*	*y*	*z*	*U*_iso_*/*U*_eq_	Occ. (<1)
Ta1	0.64607 (2)	−0.03553 (2)	1.12174 (2)	0.01205 (4)	
Ta2	0.63326 (2)	0.13631 (2)	0.89100 (2)	0.01300 (4)	
Ta3	0.37445 (2)	0.14420 (2)	1.07511 (2)	0.01322 (4)	
O1	0.7572 (2)	−0.0558 (2)	1.21206 (18)	0.0165 (5)	
O2	0.7303 (2)	0.2421 (2)	0.8064 (2)	0.0217 (5)	
O3	0.2829 (3)	0.2550 (2)	1.1292 (2)	0.0234 (6)	
O4	0.7273 (2)	0.0788 (2)	1.00901 (19)	0.0147 (5)	
O5	0.4815 (2)	0.1550 (2)	0.81001 (18)	0.0158 (5)	
O6	0.7149 (2)	−0.1593 (2)	1.04715 (19)	0.0149 (5)	
O7	0.5176 (2)	0.0863 (2)	1.15884 (19)	0.0164 (5)	
O8	0.7070 (2)	−0.0048 (2)	0.85123 (18)	0.0159 (5)	
O9	0.5036 (2)	0.2344 (2)	0.96735 (19)	0.0156 (5)	
O10	0.500000	0.000000	1.000000	0.0136 (7)	
Ni1	0.92251 (4)	−0.03103 (4)	1.26360 (3)	0.01532 (9)	
N1	0.8699 (3)	0.1472 (3)	1.2539 (2)	0.0201 (6)	
C1	0.8585 (4)	0.2037 (3)	1.1455 (3)	0.0230 (8)	
H1A	0.853129	0.288863	1.133133	0.028*	
H1B	0.777943	0.185909	1.127343	0.028*	
C2	0.9754 (4)	0.1607 (4)	1.0799 (3)	0.0270 (8)	
H2A	0.960794	0.188815	1.007761	0.032*	
H2B	1.053812	0.191245	1.088285	0.032*	
N2	0.9951 (3)	0.0334 (3)	1.1095 (2)	0.0211 (6)	
H2C	1.081340	0.008218	1.099923	0.025*	
H2D	0.953545	0.005768	1.069973	0.025*	
C3	0.9730 (4)	0.1919 (4)	1.2894 (3)	0.0292 (9)	
H3A	0.931892	0.250220	1.326166	0.035*	
H3B	1.029801	0.231278	1.229806	0.035*	
C4	1.0561 (4)	0.0973 (4)	1.3584 (3)	0.0253 (8)	
H4A	1.137072	0.126636	1.363251	0.030*	
H4B	1.008365	0.075941	1.427187	0.030*	
N3	1.0883 (3)	−0.0053 (3)	1.3184 (2)	0.0197 (6)	
H3C	1.113045	−0.068663	1.368206	0.024*	
H3D	1.155159	0.005610	1.267602	0.024*	
C5	0.7429 (4)	0.1552 (4)	1.3166 (3)	0.0257 (8)	
H5A	0.674670	0.141584	1.280900	0.031*	
H5B	0.721195	0.234233	1.326618	0.031*	
C6	0.7451 (4)	0.0670 (4)	1.4187 (3)	0.0268 (8)	
H6A	0.798205	0.090586	1.460793	0.032*	
H6B	0.655662	0.063614	1.454163	0.032*	
N4	0.8004 (3)	−0.0488 (3)	1.4047 (2)	0.0234 (7)	
H4C	0.734953	−0.090387	1.405737	0.028*	
H4D	0.847421	−0.087973	1.456312	0.028*	
Ni2	0.37645 (4)	0.30477 (4)	0.85661 (4)	0.01769 (9)	
N11	0.2586 (3)	0.3909 (3)	0.7352 (3)	0.0228 (6)	
C11	0.3553 (4)	0.4394 (4)	0.6485 (4)	0.0384 (11)	
H11A	0.308709	0.496041	0.595074	0.046*	
H11B	0.401692	0.375854	0.619627	0.046*	
C12	0.4526 (5)	0.4980 (4)	0.6785 (4)	0.0476 (14)	
H12A	0.524285	0.514673	0.622820	0.057*	
H12B	0.410697	0.572483	0.691769	0.057*	
N12	0.5045 (4)	0.4215 (3)	0.7703 (3)	0.0384 (10)	
H12C	0.521448	0.465760	0.809050	0.046*	
H12D	0.580819	0.380862	0.752063	0.046*	
C13	0.1726 (4)	0.4869 (3)	0.7696 (3)	0.0282 (8)	
H13A	0.092034	0.504736	0.736649	0.034*	
H13B	0.217298	0.557486	0.748922	0.034*	
C14	0.1381 (4)	0.4544 (3)	0.8830 (3)	0.0260 (8)	
H14A	0.087344	0.521228	0.905006	0.031*	
H14B	0.084937	0.389170	0.903890	0.031*	
N13	0.2613 (3)	0.4200 (3)	0.9300 (3)	0.0247 (7)	
H13C	0.244850	0.385428	0.997304	0.030*	
H13D	0.302153	0.483347	0.922462	0.030*	
C15	0.1831 (4)	0.3213 (3)	0.6974 (3)	0.0222 (7)	
H15A	0.241988	0.254676	0.681442	0.027*	
H15B	0.150923	0.369778	0.633641	0.027*	
C16	0.0684 (3)	0.2759 (3)	0.7707 (3)	0.0197 (7)	
H16A	0.098349	0.228688	0.835662	0.024*	
H16B	0.005448	0.341548	0.784192	0.024*	
N14	0.0054 (3)	0.2040 (3)	0.7259 (2)	0.0202 (6)	
H14C	0.004942	0.240492	0.658896	0.024*	
H14D	−0.079034	0.205534	0.754004	0.024*	
O11	0.8066 (3)	0.3844 (3)	0.9091 (3)	0.0378 (7)	
H11C	0.779756	0.353130	0.870404	0.057*	
H11D	0.733546	0.387800	0.943614	0.057*	
O12	1.4219 (4)	0.0650 (4)	1.3614 (3)	0.0536 (10)	
H12E	1.450539	0.085993	1.299839	0.080*	
H12F	1.428029	−0.007777	1.376809	0.080*	
O13	−0.1468 (4)	0.5884 (3)	0.7766 (3)	0.0537 (11)	
H13E	−0.177526	0.528101	0.812709	0.081*	
H13F	−0.198956	0.645501	0.786419	0.081*	
O14	0.5770 (3)	0.3988 (3)	1.0444 (3)	0.0459 (9)	
H14E	0.550254	0.380175	1.106447	0.069*	
H14F	0.528754	0.354705	1.034817	0.069*	
O15	0.6112 (4)	−0.2362 (3)	1.5414 (3)	0.0511 (9)	
H15C	0.683268	−0.271482	1.557895	0.077*	
H15D	0.595218	−0.177272	1.563995	0.077*	
O16	1.4662 (9)	−0.1666 (9)	1.3937 (7)	0.0297 (19)	0.5
O16'	1.4319 (10)	−0.1976 (9)	1.3891 (8)	0.0316 (19)	0.5
O17	−0.1862 (9)	0.5860 (7)	0.5871 (6)	0.0354 (18)	0.5
O17'	−0.1465 (9)	0.6271 (8)	0.5822 (8)	0.042 (2)	0.5
O18	1.2376 (6)	−0.2566 (6)	1.4074 (4)	0.0351 (14)	0.5
O18'	1.2585 (6)	−0.3647 (6)	1.3875 (5)	0.0398 (16)	0.5
O19	−0.0677 (7)	0.4198 (6)	0.5130 (5)	0.0403 (15)	0.5
O19'	1.0608 (8)	−0.2496 (6)	1.4918 (5)	0.0445 (17)	0.5
O20	0.5580 (6)	0.6592 (5)	0.7647 (4)	0.0285 (12)	0.5

### Atomic displacement parameters (Å^2^)

**Table:**

	*U*^11^	*U*^22^	*U*^33^	*U*^12^	*U*^13^	*U*^23^
Ta1	0.00591 (6)	0.01739 (7)	0.01444 (7)	−0.00135 (5)	−0.00280 (5)	−0.00589 (5)
Ta2	0.00604 (6)	0.01679 (7)	0.01625 (7)	−0.00248 (5)	−0.00202 (5)	−0.00348 (5)
Ta3	0.00737 (7)	0.01741 (7)	0.01720 (7)	−0.00015 (5)	−0.00273 (5)	−0.00837 (5)
O1	0.0090 (10)	0.0255 (13)	0.0164 (11)	−0.0017 (9)	−0.0042 (9)	−0.0066 (10)
O2	0.0119 (11)	0.0221 (13)	0.0267 (14)	−0.0055 (10)	−0.0021 (10)	0.0025 (10)
O3	0.0181 (13)	0.0254 (14)	0.0306 (15)	0.0042 (10)	−0.0039 (11)	−0.0168 (12)
O4	0.0074 (10)	0.0169 (11)	0.0214 (12)	−0.0026 (8)	−0.0033 (9)	−0.0061 (9)
O5	0.0092 (11)	0.0213 (12)	0.0160 (11)	−0.0015 (9)	−0.0029 (9)	−0.0027 (9)
O6	0.0068 (10)	0.0188 (12)	0.0204 (12)	−0.0006 (9)	−0.0035 (9)	−0.0065 (9)
O7	0.0117 (11)	0.0225 (12)	0.0186 (12)	−0.0007 (9)	−0.0040 (9)	−0.0108 (10)
O8	0.0079 (10)	0.0237 (12)	0.0169 (12)	−0.0019 (9)	−0.0003 (9)	−0.0071 (10)
O9	0.0109 (11)	0.0147 (11)	0.0230 (12)	−0.0017 (9)	−0.0054 (9)	−0.0060 (10)
O10	0.0076 (15)	0.0186 (17)	0.0148 (16)	0.0005 (12)	−0.0037 (12)	−0.0045 (13)
Ni1	0.00831 (18)	0.0236 (2)	0.0161 (2)	−0.00135 (16)	−0.00322 (15)	−0.00786 (17)
N1	0.0167 (14)	0.0240 (15)	0.0215 (15)	−0.0018 (11)	−0.0066 (12)	−0.0070 (12)
C1	0.0186 (17)	0.0240 (18)	0.0272 (19)	−0.0046 (14)	−0.0089 (15)	−0.0035 (15)
C2	0.0162 (17)	0.037 (2)	0.0244 (19)	−0.0087 (15)	−0.0005 (14)	−0.0015 (16)
N2	0.0090 (13)	0.0366 (17)	0.0192 (14)	−0.0031 (12)	−0.0028 (11)	−0.0089 (13)
C3	0.028 (2)	0.028 (2)	0.039 (2)	−0.0059 (16)	−0.0168 (17)	−0.0109 (17)
C4	0.0206 (18)	0.033 (2)	0.0275 (19)	−0.0053 (15)	−0.0093 (15)	−0.0126 (16)
N3	0.0104 (13)	0.0309 (17)	0.0186 (14)	−0.0041 (12)	−0.0021 (11)	−0.0068 (12)
C5	0.0177 (17)	0.031 (2)	0.030 (2)	0.0053 (15)	−0.0036 (15)	−0.0151 (17)
C6	0.0197 (18)	0.041 (2)	0.0228 (18)	−0.0021 (16)	0.0011 (14)	−0.0165 (17)
N4	0.0199 (15)	0.0321 (18)	0.0201 (15)	−0.0046 (13)	−0.0021 (12)	−0.0093 (13)
Ni2	0.0109 (2)	0.0159 (2)	0.0260 (2)	−0.00212 (16)	−0.00576 (17)	−0.00300 (18)
N11	0.0168 (14)	0.0200 (15)	0.0290 (17)	−0.0044 (12)	−0.0038 (12)	−0.0004 (13)
C11	0.023 (2)	0.037 (2)	0.040 (3)	−0.0050 (17)	−0.0006 (18)	0.013 (2)
C12	0.027 (2)	0.034 (2)	0.065 (3)	−0.0093 (19)	−0.013 (2)	0.020 (2)
N12	0.0217 (17)	0.0235 (18)	0.060 (3)	−0.0055 (14)	−0.0129 (17)	0.0115 (17)
C13	0.0221 (18)	0.0206 (18)	0.041 (2)	−0.0010 (14)	−0.0138 (17)	−0.0023 (16)
C14	0.0184 (17)	0.0202 (18)	0.041 (2)	0.0011 (14)	−0.0110 (16)	−0.0091 (16)
N13	0.0204 (15)	0.0174 (15)	0.0394 (19)	0.0006 (12)	−0.0121 (14)	−0.0093 (13)
C15	0.0183 (17)	0.0265 (19)	0.0201 (17)	−0.0004 (14)	−0.0057 (14)	−0.0030 (14)
C16	0.0144 (16)	0.0237 (18)	0.0233 (18)	0.0009 (13)	−0.0061 (13)	−0.0094 (14)
N14	0.0122 (13)	0.0271 (16)	0.0231 (15)	0.0001 (11)	−0.0059 (11)	−0.0089 (13)
O11	0.0335 (17)	0.0356 (17)	0.0454 (19)	−0.0087 (13)	0.0052 (14)	−0.0152 (14)
O12	0.061 (2)	0.077 (3)	0.0222 (16)	−0.006 (2)	0.0038 (16)	−0.0188 (17)
O13	0.085 (3)	0.0301 (17)	0.042 (2)	−0.0068 (18)	0.0191 (19)	−0.0177 (15)
O14	0.0352 (18)	0.058 (2)	0.058 (2)	−0.0262 (16)	0.0025 (16)	−0.0311 (18)
O15	0.0348 (19)	0.0385 (19)	0.075 (3)	−0.0001 (15)	−0.0046 (18)	−0.0100 (18)
O16	0.026 (5)	0.048 (6)	0.018 (3)	−0.007 (3)	−0.002 (3)	−0.013 (3)
O16'	0.035 (5)	0.037 (5)	0.025 (4)	−0.002 (3)	−0.006 (4)	−0.012 (3)
O17	0.043 (5)	0.035 (5)	0.027 (3)	0.006 (3)	−0.001 (3)	−0.014 (3)
O17'	0.035 (5)	0.047 (6)	0.051 (5)	−0.001 (4)	−0.005 (4)	−0.029 (4)
O18	0.035 (3)	0.047 (4)	0.023 (3)	−0.009 (3)	−0.009 (2)	−0.004 (3)
O18'	0.037 (4)	0.050 (4)	0.024 (3)	0.010 (3)	−0.003 (3)	−0.004 (3)
O19	0.039 (4)	0.039 (4)	0.038 (4)	−0.003 (3)	0.005 (3)	−0.008 (3)
O19'	0.061 (5)	0.040 (4)	0.034 (3)	0.008 (3)	−0.005 (3)	−0.019 (3)
O20	0.026 (3)	0.037 (3)	0.029 (3)	−0.008 (2)	−0.005 (2)	−0.016 (2)

### Geometric parameters (Å, º)

**Table:**

Ta1—Ta2	3.3013 (2)	N3—H3D	0.9100
Ta1—Ta3	3.3325 (2)	C5—H5A	0.9900
Ta1—O1	1.786 (2)	C5—H5B	0.9900
Ta1—O4	1.957 (2)	C5—C6	1.517 (6)
Ta1—O5^i^	2.057 (2)	C6—H6A	0.9900
Ta1—O6	2.030 (2)	C6—H6B	0.9900
Ta1—O7	1.957 (2)	C6—N4	1.482 (5)
Ta1—O10	2.3641 (1)	N4—H4C	0.9100
Ta2—Ta3^i^	3.3056 (2)	N4—H4D	0.9100
Ta2—O2	1.803 (3)	Ni2—N11	2.172 (3)
Ta2—O4	1.947 (2)	Ni2—N12	2.094 (4)
Ta2—O5	2.040 (2)	Ni2—N13	2.076 (3)
Ta2—O8	1.947 (2)	N11—C11	1.493 (5)
Ta2—O9	2.029 (2)	N11—C13	1.494 (5)
Ta2—O10	2.3737 (1)	N11—C15	1.484 (5)
Ta2—Ni2	3.1254 (4)	C11—H11A	0.9900
Ta3—O3	1.791 (3)	C11—H11B	0.9900
Ta3—O6^i^	2.015 (2)	C11—C12	1.501 (7)
Ta3—O7	1.964 (2)	C12—H12A	0.9900
Ta3—O8^i^	1.959 (3)	C12—H12B	0.9900
Ta3—O9	2.045 (3)	C12—N12	1.475 (6)
Ta3—O10	2.3912 (1)	N12—H12C	0.9100
Ta3—Ni2	3.1064 (5)	N12—H12D	0.9100
O1—Ni1	2.072 (2)	C13—H13A	0.9900
O5—Ni2	2.170 (3)	C13—H13B	0.9900
O6—Ni2^i^	2.149 (2)	C13—C14	1.509 (6)
O9—Ni2	2.103 (2)	C14—H14A	0.9900
Ni1—N1	2.111 (3)	C14—H14B	0.9900
Ni1—N2	2.120 (3)	C14—N13	1.489 (5)
Ni1—N3	2.105 (3)	N13—H13C	0.9100
Ni1—N4	2.139 (3)	N13—H13D	0.9100
Ni1—N14^i^	2.082 (3)	C15—H15A	0.9900
N1—C1	1.482 (5)	C15—H15B	0.9900
N1—C3	1.486 (5)	C15—C16	1.516 (5)
N1—C5	1.478 (5)	C16—H16A	0.9900
C1—H1A	0.9900	C16—H16B	0.9900
C1—H1B	0.9900	C16—N14	1.482 (5)
C1—C2	1.522 (6)	N14—H14C	0.9100
C2—H2A	0.9900	N14—H14D	0.9100
C2—H2B	0.9900	O11—H11C	0.8399
C2—N2	1.467 (5)	O11—H11D	0.8396
N2—H2C	0.9100	O12—H12E	0.8400
N2—H2D	0.9100	O12—H12F	0.8401
C3—H3A	0.9900	O13—H13E	0.8402
C3—H3B	0.9900	O13—H13F	0.8399
C3—C4	1.523 (5)	O14—H14E	0.8400
C4—H4A	0.9900	O14—H14F	0.8400
C4—H4B	0.9900	O15—H15C	0.8398
C4—N3	1.468 (5)	O15—H15D	0.8399
N3—H3C	0.9100		
			
Ta2—Ta1—Ta3	62.304 (4)	C5—N1—C3	113.4 (3)
O1—Ta1—Ta2	132.06 (8)	N1—C1—H1A	109.6
O1—Ta1—Ta3	132.68 (8)	N1—C1—H1B	109.6
O1—Ta1—O4	99.95 (11)	N1—C1—C2	110.2 (3)
O1—Ta1—O5^i^	104.23 (11)	H1A—C1—H1B	108.1
O1—Ta1—O6	104.25 (10)	C2—C1—H1A	109.6
O1—Ta1—O7	100.84 (11)	C2—C1—H1B	109.6
O1—Ta1—O10	177.50 (8)	C1—C2—H2A	109.8
O4—Ta1—Ta2	32.17 (7)	C1—C2—H2B	109.8
O4—Ta1—Ta3	84.14 (7)	H2A—C2—H2B	108.2
O4—Ta1—O5^i^	155.30 (10)	N2—C2—C1	109.5 (3)
O4—Ta1—O6	89.21 (10)	N2—C2—H2A	109.8
O4—Ta1—O7	90.86 (10)	N2—C2—H2B	109.8
O4—Ta1—O10	78.10 (7)	Ni1—N2—H2C	109.5
O5^i^—Ta1—Ta2	123.66 (7)	Ni1—N2—H2D	109.5
O5^i^—Ta1—Ta3	83.23 (7)	C2—N2—Ni1	110.6 (2)
O5^i^—Ta1—O10	77.86 (7)	C2—N2—H2C	109.5
O6—Ta1—Ta2	83.44 (7)	C2—N2—H2D	109.5
O6—Ta1—Ta3	123.02 (7)	H2C—N2—H2D	108.1
O6—Ta1—O5^i^	80.09 (10)	N1—C3—H3A	108.9
O6—Ta1—O10	77.37 (7)	N1—C3—H3B	108.9
O7—Ta1—Ta2	83.14 (7)	N1—C3—C4	113.3 (3)
O7—Ta1—Ta3	31.87 (7)	H3A—C3—H3B	107.7
O7—Ta1—O5^i^	89.53 (10)	C4—C3—H3A	108.9
O7—Ta1—O6	154.51 (10)	C4—C3—H3B	108.9
O7—Ta1—O10	77.71 (7)	C3—C4—H4A	109.6
O10—Ta1—Ta2	45.949 (3)	C3—C4—H4B	109.6
O10—Ta1—Ta3	45.842 (3)	H4A—C4—H4B	108.1
Ta1—Ta2—Ta3^i^	61.789 (4)	N3—C4—C3	110.1 (3)
O2—Ta2—Ta1	135.83 (8)	N3—C4—H4A	109.6
O2—Ta2—Ta3^i^	134.41 (9)	N3—C4—H4B	109.6
O2—Ta2—O4	103.52 (11)	Ni1—N3—H3C	110.0
O2—Ta2—O5	100.46 (11)	Ni1—N3—H3D	110.0
O2—Ta2—O8	102.12 (12)	C4—N3—Ni1	108.3 (2)
O2—Ta2—O9	102.38 (11)	C4—N3—H3C	110.0
O2—Ta2—O10	178.28 (8)	C4—N3—H3D	110.0
O2—Ta2—Ni2	92.21 (8)	H3C—N3—H3D	108.4
O4—Ta2—Ta1	32.35 (7)	N1—C5—H5A	109.4
O4—Ta2—Ta3^i^	81.68 (7)	N1—C5—H5B	109.4
O4—Ta2—O5	155.76 (10)	N1—C5—C6	111.1 (3)
O4—Ta2—O8	87.94 (10)	H5A—C5—H5B	108.0
O4—Ta2—O9	89.28 (10)	C6—C5—H5A	109.4
O4—Ta2—O10	78.04 (7)	C6—C5—H5B	109.4
O4—Ta2—Ni2	130.95 (7)	C5—C6—H6A	109.7
O5—Ta2—Ta1	123.50 (7)	C5—C6—H6B	109.7
O5—Ta2—Ta3^i^	84.17 (7)	H6A—C6—H6B	108.2
O5—Ta2—O10	77.94 (7)	N4—C6—C5	109.8 (3)
O5—Ta2—Ni2	43.70 (7)	N4—C6—H6A	109.7
O8—Ta2—Ta1	83.13 (7)	N4—C6—H6B	109.7
O8—Ta2—Ta3^i^	32.29 (7)	Ni1—N4—H4C	109.6
O8—Ta2—O5	90.54 (10)	Ni1—N4—H4D	109.6
O8—Ta2—O9	155.32 (10)	C6—N4—Ni1	110.2 (2)
O8—Ta2—O10	78.58 (7)	C6—N4—H4C	109.6
O8—Ta2—Ni2	134.06 (7)	C6—N4—H4D	109.6
O9—Ta2—Ta1	81.47 (7)	H4C—N4—H4D	108.1
O9—Ta2—Ta3^i^	123.11 (7)	Ta3—Ni2—Ta2	66.828 (9)
O9—Ta2—O5	82.10 (10)	O5—Ni2—Ta2	40.50 (6)
O9—Ta2—O10	76.85 (7)	O5—Ni2—Ta3	85.33 (6)
O9—Ta2—Ni2	41.73 (7)	O5—Ni2—N11	103.85 (11)
O10—Ta2—Ta1	45.710 (3)	O6^i^—Ni2—Ta2	84.09 (6)
O10—Ta2—Ta3^i^	46.292 (3)	O6^i^—Ni2—Ta3	40.14 (6)
O10—Ta2—Ni2	86.199 (9)	O6^i^—Ni2—O5	74.98 (9)
Ni2—Ta2—Ta1	115.605 (10)	O6^i^—Ni2—N11	108.66 (11)
Ni2—Ta2—Ta3^i^	118.719 (9)	O9—Ni2—Ta2	39.97 (7)
Ta2^i^—Ta3—Ta1	61.584 (4)	O9—Ni2—Ta3	40.81 (7)
O3—Ta3—Ta1	134.81 (9)	O9—Ni2—O5	77.40 (10)
O3—Ta3—Ta2^i^	135.63 (9)	O9—Ni2—O6^i^	77.27 (10)
O3—Ta3—O6^i^	102.86 (11)	O9—Ni2—N11	174.07 (11)
O3—Ta3—O7	103.09 (11)	N11—Ni2—Ta2	139.05 (9)
O3—Ta3—O8^i^	103.56 (12)	N11—Ni2—Ta3	144.70 (8)
O3—Ta3—O9	102.36 (12)	N12—Ni2—Ta2	82.86 (10)
O3—Ta3—O10	178.51 (10)	N12—Ni2—Ta3	131.62 (11)
O3—Ta3—Ni2	92.55 (9)	N12—Ni2—O5	95.51 (14)
O6^i^—Ta3—Ta1	121.97 (7)	N12—Ni2—O6^i^	166.92 (12)
O6^i^—Ta3—Ta2^i^	83.52 (7)	N12—Ni2—O9	92.05 (13)
O6^i^—Ta3—O9	81.65 (10)	N12—Ni2—N11	82.07 (13)
O6^i^—Ta3—O10	76.99 (7)	N13—Ni2—Ta2	136.14 (9)
O6^i^—Ta3—Ni2	43.43 (7)	N13—Ni2—Ta3	82.71 (10)
O7—Ta3—Ta1	31.73 (7)	N13—Ni2—O5	167.02 (12)
O7—Ta3—Ta2^i^	82.02 (7)	N13—Ni2—O6^i^	92.56 (12)
O7—Ta3—O6^i^	153.41 (10)	N13—Ni2—O9	96.59 (12)
O7—Ta3—O9	87.26 (10)	N13—Ni2—N11	83.33 (13)
O7—Ta3—O10	76.91 (7)	N13—Ni2—N12	96.20 (16)
O7—Ta3—Ni2	129.41 (7)	C11—N11—Ni2	103.7 (2)
O8^i^—Ta3—Ta1	82.41 (7)	C11—N11—C13	110.2 (3)
O8^i^—Ta3—Ta2^i^	32.08 (7)	C13—N11—Ni2	105.8 (2)
O8^i^—Ta3—O6^i^	91.15 (10)	C15—N11—Ni2	119.3 (2)
O8^i^—Ta3—O7	88.35 (11)	C15—N11—C11	106.1 (3)
O8^i^—Ta3—O9	154.04 (10)	C15—N11—C13	111.3 (3)
O8^i^—Ta3—O10	77.93 (7)	N11—C11—H11A	109.0
O8^i^—Ta3—Ni2	134.43 (7)	N11—C11—H11B	109.0
O9—Ta3—Ta1	80.45 (7)	N11—C11—C12	112.7 (4)
O9—Ta3—Ta2^i^	121.97 (7)	H11A—C11—H11B	107.8
O9—Ta3—O10	76.15 (7)	C12—C11—H11A	109.0
O9—Ta3—Ni2	42.21 (7)	C12—C11—H11B	109.0
O10—Ta3—Ta1	45.177 (3)	C11—C12—H12A	109.8
O10—Ta3—Ta2^i^	45.855 (3)	C11—C12—H12B	109.8
O10—Ta3—Ni2	86.339 (9)	H12A—C12—H12B	108.3
Ni2—Ta3—Ta1	115.241 (9)	N12—C12—C11	109.2 (4)
Ni2—Ta3—Ta2^i^	118.381 (9)	N12—C12—H12A	109.8
Ta1—O1—Ni1	155.51 (15)	N12—C12—H12B	109.8
Ta2—O4—Ta1	115.48 (11)	Ni2—N12—H12C	109.0
Ta1^i^—O5—Ni2	99.62 (10)	Ni2—N12—H12D	109.0
Ta2—O5—Ta1^i^	112.10 (11)	C12—N12—Ni2	112.7 (3)
Ta2—O5—Ni2	95.80 (10)	C12—N12—H12C	109.0
Ta1—O6—Ni2^i^	101.20 (10)	C12—N12—H12D	109.0
Ta3^i^—O6—Ta1	114.00 (11)	H12C—N12—H12D	107.8
Ta3^i^—O6—Ni2^i^	96.43 (10)	N11—C13—H13A	109.4
Ta1—O7—Ta3	116.40 (12)	N11—C13—H13B	109.4
Ta2—O8—Ta3^i^	115.63 (12)	N11—C13—C14	111.3 (3)
Ta2—O9—Ta3	114.78 (11)	H13A—C13—H13B	108.0
Ta2—O9—Ni2	98.30 (11)	C14—C13—H13A	109.4
Ta3—O9—Ni2	96.99 (10)	C14—C13—H13B	109.4
Ta1—O10—Ta1^i^	180.0	C13—C14—H14A	110.1
Ta1—O10—Ta2	88.341 (5)	C13—C14—H14B	110.1
Ta1^i^—O10—Ta2^i^	88.341 (5)	H14A—C14—H14B	108.4
Ta1^i^—O10—Ta2	91.659 (4)	N13—C14—C13	107.9 (3)
Ta1—O10—Ta2^i^	91.659 (4)	N13—C14—H14A	110.1
Ta1—O10—Ta3	88.980 (4)	N13—C14—H14B	110.1
Ta1—O10—Ta3^i^	91.020 (5)	Ni2—N13—H13C	110.4
Ta1^i^—O10—Ta3	91.020 (4)	Ni2—N13—H13D	110.4
Ta1^i^—O10—Ta3^i^	88.980 (4)	C14—N13—Ni2	106.8 (2)
Ta2^i^—O10—Ta2	180.0	C14—N13—H13C	110.4
Ta2^i^—O10—Ta3	87.852 (5)	C14—N13—H13D	110.4
Ta2^i^—O10—Ta3^i^	92.147 (5)	H13C—N13—H13D	108.6
Ta2—O10—Ta3^i^	87.854 (5)	N11—C15—H15A	108.7
Ta2—O10—Ta3	92.147 (5)	N11—C15—H15B	108.7
Ta3—O10—Ta3^i^	180.0	N11—C15—C16	114.4 (3)
O1—Ni1—N1	96.31 (11)	H15A—C15—H15B	107.6
O1—Ni1—N2	86.87 (11)	C16—C15—H15A	108.7
O1—Ni1—N3	178.92 (11)	C16—C15—H15B	108.7
O1—Ni1—N4	83.49 (11)	C15—C16—H16A	109.9
O1—Ni1—N14^i^	89.60 (11)	C15—C16—H16B	109.9
N1—Ni1—N2	82.40 (13)	H16A—C16—H16B	108.3
N1—Ni1—N4	82.30 (13)	N14—C16—C15	108.9 (3)
N2—Ni1—N4	160.88 (12)	N14—C16—H16A	109.9
N3—Ni1—N1	83.27 (12)	N14—C16—H16B	109.9
N3—Ni1—N2	94.05 (12)	Ni1^i^—N14—H14C	107.1
N3—Ni1—N4	95.46 (12)	Ni1^i^—N14—H14D	107.1
N14^i^—Ni1—N1	173.85 (12)	C16—N14—Ni1^i^	120.8 (2)
N14^i^—Ni1—N2	96.26 (13)	C16—N14—H14C	107.1
N14^i^—Ni1—N3	90.85 (12)	C16—N14—H14D	107.1
N14^i^—Ni1—N4	100.14 (13)	H14C—N14—H14D	106.8
C1—N1—Ni1	105.6 (2)	H11C—O11—H11D	92.0
C1—N1—C3	111.1 (3)	H12E—O12—H12F	106.4
C3—N1—Ni1	109.3 (2)	H13E—O13—H13F	107.5
C5—N1—Ni1	105.5 (2)	H14E—O14—H14F	89.4
C5—N1—C1	111.5 (3)	H15C—O15—H15D	108.4
			
Ta2—Ta1—O1—Ni1	−5.5 (4)	C5—N1—C1—C2	−161.0 (3)
Ta3—Ta1—O1—Ni1	−94.3 (3)	C5—N1—C3—C4	95.1 (4)
O4—Ta1—O1—Ni1	−3.1 (4)	C5—C6—N4—Ni1	24.0 (4)
O5^i^—Ta1—O1—Ni1	171.7 (3)	Ni2—N11—C11—C12	−45.1 (4)
O6—Ta1—O1—Ni1	88.6 (4)	Ni2—N11—C13—C14	−32.3 (3)
O7—Ta1—O1—Ni1	−96.0 (3)	Ni2—N11—C15—C16	70.5 (4)
Ni1—N1—C1—C2	−46.8 (3)	N11—C11—C12—N12	48.2 (6)
Ni1—N1—C3—C4	−22.4 (4)	N11—C13—C14—N13	55.0 (4)
Ni1—N1—C5—C6	47.6 (3)	N11—C15—C16—N14	−177.5 (3)
N1—C1—C2—N2	50.5 (4)	C11—N11—C13—C14	−143.9 (3)
N1—C3—C4—N3	42.7 (5)	C11—N11—C15—C16	−173.1 (3)
N1—C5—C6—N4	−48.8 (4)	C11—C12—N12—Ni2	−24.9 (6)
C1—N1—C3—C4	−138.5 (4)	C13—N11—C11—C12	67.8 (4)
C1—N1—C5—C6	161.7 (3)	C13—N11—C15—C16	−53.2 (4)
C1—C2—N2—Ni1	−27.3 (4)	C13—C14—N13—Ni2	−48.0 (3)
C3—N1—C1—C2	71.5 (4)	C15—N11—C11—C12	−171.6 (4)
C3—N1—C5—C6	−72.0 (4)	C15—N11—C13—C14	98.6 (4)
C3—C4—N3—Ni1	−40.5 (4)	C15—C16—N14—Ni1^i^	82.7 (3)

Symmetry code: (i) −*x*+1, −*y*, −*z*+2.

### Hydrogen-bond geometry (Å, º)

**Table:**

*D*—H···*A*	*D*—H	H···*A*	*D*···*A*	*D*—H···*A*
C1—H1*B*···O4	0.99	2.52	3.293 (5)	135
N2—H2*C*···O4^ii^	0.91	2.59	3.394 (4)	148
N2—H2*C*···O8^ii^	0.91	2.42	3.208 (4)	146
N2—H2*D*···O4	0.91	2.59	3.236 (4)	129
N2—H2*D*···N2^ii^	0.91	2.62	3.338 (6)	136
C3—H3*A*···O19′^iii^	0.99	2.53	3.253 (8)	129
C3—H3*B*···O13^iv^	0.99	2.64	3.301 (6)	124
C4—H4*B*···O19′^iii^	0.99	2.60	3.135 (7)	114
N3—H3*C*···O18	0.91	2.40	3.197 (7)	146
N3—H3*C*···O19′	0.91	2.45	3.266 (8)	149
N3—H3*D*···O8^ii^	0.91	2.02	2.915 (4)	169
N4—H4*C*···O15	0.91	2.54	3.244 (5)	135
C11—H11*B*···O16′^ii^	0.99	2.57	3.497 (12)	156
C12—H12*B*···O20	0.99	2.47	2.977 (8)	111
N12—H12*C*···O20	0.91	2.33	2.995 (7)	130
N12—H12*D*···O2	0.91	2.16	2.951 (4)	145
N12—H12*D*···O18′^ii^	0.91	2.39	3.168 (8)	144
C13—H13*A*···O13	0.99	2.59	3.380 (6)	137
N13—H13*C*···O3	0.91	2.10	2.937 (5)	153
N13—H13*D*···O14^iv^	0.91	2.23	3.103 (5)	160
C15—H15*A*···O1^i^	0.99	2.45	3.105 (5)	124
C16—H16*A*···O6^i^	0.99	2.63	3.514 (4)	149
C16—H16*B*···O11^v^	0.99	2.58	3.400 (5)	140
N14—H14*C*···O19	0.91	2.62	3.447 (7)	151
N14—H14*C*···O19′^i^	0.91	2.27	3.082 (7)	148
N14—H14*D*···O2^v^	0.91	2.04	2.941 (4)	169
O11—H11*C*···O2	0.84	1.97	2.794 (4)	165
O11—H11*D*···O14	0.84	2.00	2.826 (5)	170
O12—H12*E*···O7^vi^	0.84	1.97	2.784 (4)	163
O12—H12*F*···O16	0.84	1.86	2.686 (11)	170
O12—H12*F*···O16′	0.84	2.25	3.079 (11)	169
O13—H13*E*···O11^v^	0.84	1.89	2.696 (5)	161
O13—H13*F*···O3^vii^	0.84	1.93	2.698 (4)	152
O13—H13*F*···O20^v^	0.84	2.59	3.101 (7)	120
O14—H14*E*···O20^iv^	0.84	1.94	2.757 (7)	164
O14—H14*F*···O9	0.84	2.01	2.762 (4)	149
O15—H15*C*···O17^viii^	0.84	2.01	2.787 (9)	153
O15—H15*C*···O17′^viii^	0.84	2.02	2.850 (10)	170
O15—H15*D*···O12^iii^	0.84	1.90	2.723 (6)	168

Symmetry codes: (i) −*x*+1, −*y*, −*z*+2; (ii) −*x*+2, −*y*, −*z*+2; (iii) −*x*+2, −*y*, −*z*+3; (iv) −*x*+1, −*y*+1, −*z*+2; (v) *x*−1, *y*, *z*; (vi) *x*+1, *y*, *z*; (vii) −*x*, −*y*+1, −*z*+2; (viii) *x*+1, *y*−1, *z*+1.
