# Supplementary figures and images for: Synthesis and crystal structure of bis­[μ-N,N-bis­(2-amino­eth­yl)ethane-1,2-di­amine]­bis­[N,N-bis­(2-amino­eth­yl)ethane-1,2-di­amine]-μ4-oxido-hexa-μ3-oxido-octa-μ2-oxido-tetra­oxido­tetra­nickel(II)hexa­tantalum(V) nona­deca­hydrate
Source: Acta Crystallogr E Crystallogr Commun. 2021 Nov 9;77(Pt 12):1253–7. doi: 10.1107/S2056989021011531 (PMC8647742; doi:10.1107/S2056989021011531)

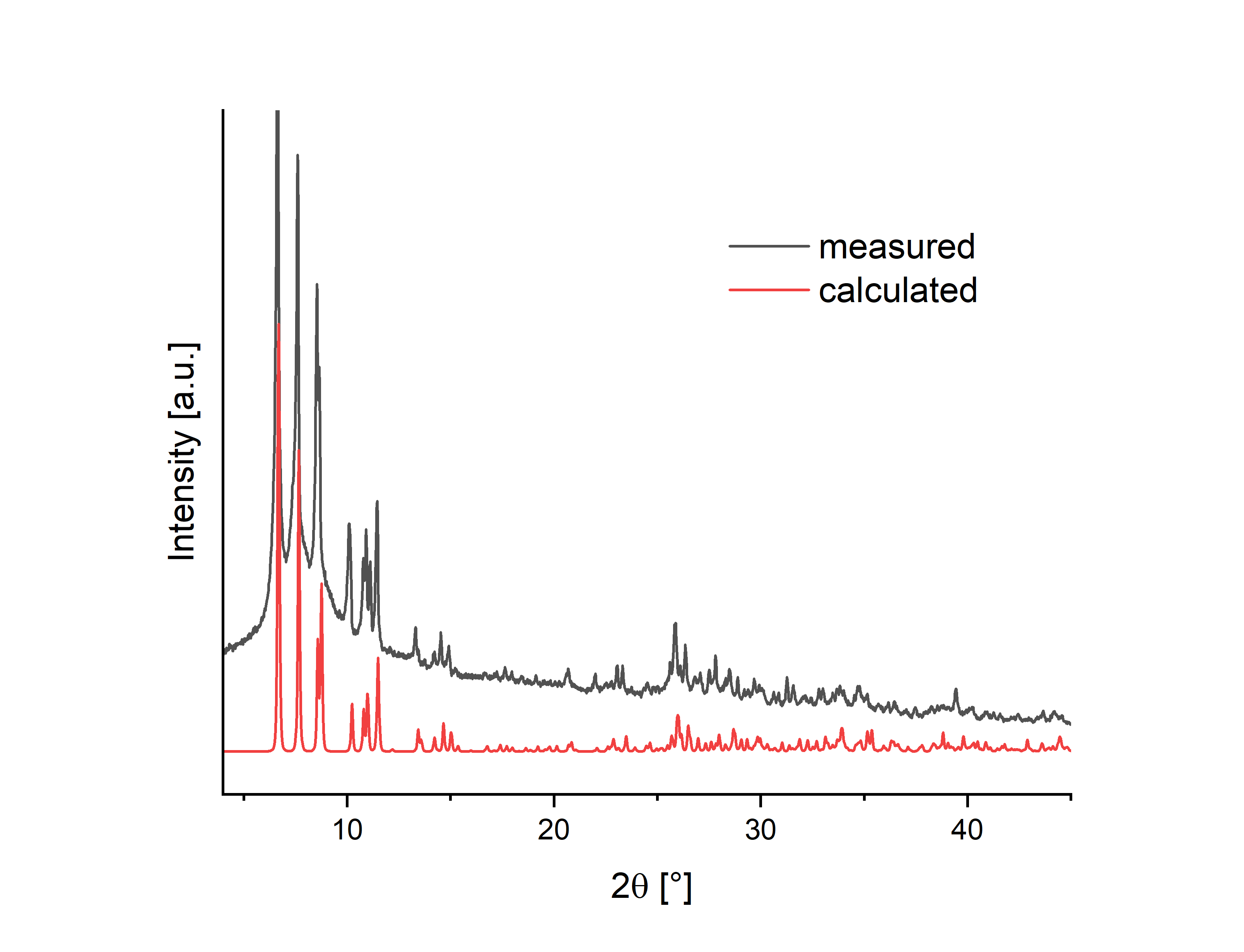

Supplement: Supplementary file 3 [file e-77-01253-sup3.tif]
